# Supplementary material for: A Comprehensive Assessment of Ultraviolet-Radiation-Induced Mutations in Flammulina filiformis Using Whole-Genome Resequencing
Source: J Fungi (Basel). 2024 Mar 20;10(3):228. doi: 10.3390/jof10030228 (PMC10971301; doi:10.3390/jof10030228)
Supplement: Supplementary file 1 [file jof-10-00228-s001.zip › Supplementary Material S8/KEGG annotation/out/64381550635650.os/KO/out_map/map04370.html]

KEGG PATHWAY: VEGF signaling pathway - Reference pathway


|  |  |
| --- | --- |
| **VEGF signaling pathway - Reference pathway** |  |

[
Pathway menu
| Organism menu
| Pathway entry
| Show description
| User data mapping
]

There is now much evidence that VEGFR-2 is the major mediator of VEGF-driven responses in endothelial cells and it is considered to be a crucial signal transducer in both physiologic and pathologic angiogenesis. The binding of VEGF to VEGFR-2 leads to a cascade of different signaling pathways, resulting in the up-regulation of genes involved in mediating the proliferation and migration of endothelial cells and promoting their survival and vascular permeability. For example, the binding of VEGF to VEGFR-2 leads to dimerization of the receptor, followed by intracellular activation of the PLCgamma;PKC-Raf kinase-MEK-mitogen-activated protein kinase (MAPK) pathway and subsequent initiation of DNA synthesis and cell growth, whereas activation of the phosphatidylinositol 3' -kinase (PI3K)-Akt pathway leads to increased endothelial-cell survival. Activation of PI3K, FAK, and p38 MAPK is implicated in cell migration signaling.

|  |  |
| --- | --- |
| Reference pathway | 100% |
